# N-Acetyl Cysteine Targets Hepatic Lipid Accumulation to Curb Oxidative Stress and Inflammation in NAFLD: A Comprehensive Analysis of the Literature

**DOI:** 10.3390/antiox9121283

**Published:** 2020-12-16

**Authors:** Phiwayinkosi V. Dludla, Bongani B. Nkambule, Sithandiwe E. Mazibuko-Mbeje, Tawanda M. Nyambuya, Fabio Marcheggiani, Ilenia Cirilli, Khanyisani Ziqubu, Samukelisiwe C. Shabalala, Rabia Johnson, Johan Louw, Elisabetta Damiani, Luca Tiano

**Affiliations:** 1 Biomedical Research and Innovation Platform, South African Medical Research Council, Tygerberg 7505, South Africa; samukelisiwe.shabalala@mrc.ac.za (S.C.S.); rabia.johnson@mrc.ac.za (R.J.); johan.louw@mrc.ac.za (J.L.); 2 Department of Life and Environmental Sciences, Polytechnic University of Marche, 60131 Ancona, Italy; f.marcheggiani@univpm.it (F.M.); ilenia.cirilli@unicam.it (I.C.); e.damiani@univpm.it (E.D.); l.tiano@univpm.it (L.T.); 3 School of Laboratory Medicine and Medical Sciences, College of Health Sciences, University of KwaZulu-Natal, Durban 4000, South Africa; nkambuleb@ukzn.ac.za (B.B.N.); mnyambuya@nust.na (T.M.N.); 4 Department of Biochemistry, Faculty of Natural and Agricultural Sciences, North-West University, Mmabatho 2745, South Africa; 36588296@nwu.ac.za (S.E.M.-M.); ziqubukhanyisani@gmail.com (K.Z.); 5 Department of Health Sciences, Faculty of Health and Applied Sciences, Namibia University of Science and Technology, Windhoek 9000, Namibia; 6 School of Pharmacy, University of Camerino, 62032 Camerino, Italy; 7 Department of Biochemistry and Microbiology, University of Zululand, KwaDlangezwa 3880, South Africa; 8 Division of Medical Physiology, Faculty of Health Sciences, Stellenbosch University, Tygerberg 7505, South Africa

**Keywords:** N-acetyl cysteine, antioxidants, non-alcoholic fatty liver disease, hepatic lipid accumulation, inflammation, oxidative stress

## Abstract

Impaired adipose tissue function and insulin resistance remain instrumental in promoting hepatic lipid accumulation in conditions of metabolic syndrome. In fact, enhanced lipid accumulation together with oxidative stress and an abnormal inflammatory response underpin the development and severity of non-alcoholic fatty liver disease (NAFLD). There are currently no specific protective drugs against NAFLD, and effective interventions involving regular exercise and healthy diets have proved difficult to achieve and maintain. Alternatively, due to its antioxidant and anti-inflammatory properties, there has been growing interest in understanding the therapeutic effects of N-acetyl cysteine (NAC) against metabolic complications, including NAFLD. Here, reviewed evidence suggests that NAC blocks hepatic lipid accumulation in preclinical models of NAFLD. This is in part through the effective regulation of a fatty acid scavenger molecule (CD36) and transcriptional factors such as sterol regulatory element-binding protein (SREBP)-1c/-2 and peroxisome proliferator-activated receptor gamma (PPARγ). Importantly, NAC appears effective in improving liver function by reducing pro-inflammatory markers such as interleukin (IL)-6 IL-1β, tumour necrosis factor alpha (TNF-α) and nuclear factor kappa-light-chain-enhancer of activated B cells (NF-κB). This was primarily through the attenuation of lipid peroxidation and enhancements in intracellular response antioxidants, particularly glutathione. Very few clinical studies support the beneficial effects of NAC against NAFLD-related complications, thus well-organized randomized clinical trials are still necessary to confirm its therapeutic potential.

## 1. Introduction

**Figure 1:**
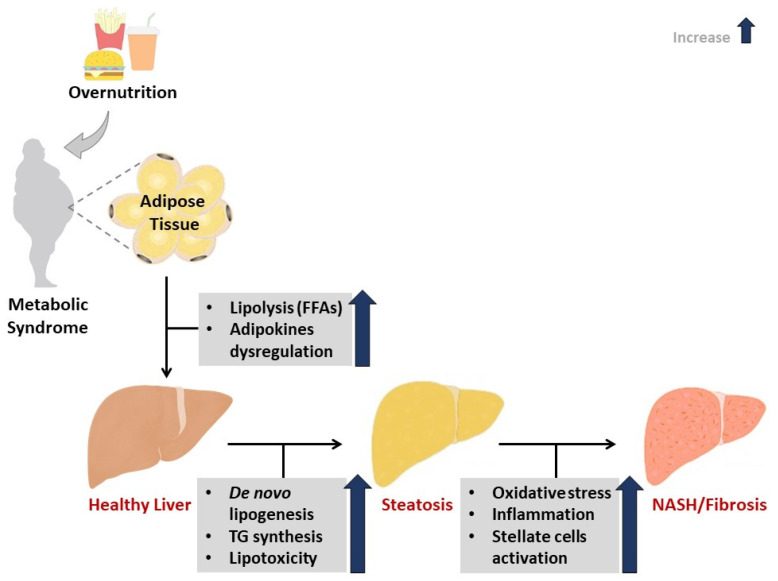
An overview of pathophysiological mechanisms that link metabolic syndrome and the development of non-alcoholic fatty liver disease (NAFLD). Briefly, impaired adipose tissue function and insulin resistance remain instrumental in promoting hepatic lipid accumulation in conditions of metabolic syndrome. In such cases, enhanced lipolysis, together with impaired adipokine function can contribute to the development of oxidative stress and an abnormal inflammatory response. The degree of oxidative stress and inflammation can define the deterioration of NAFLD, even leading to the liver steatosis and non-alcoholic steatohepatitis (NASH). TG = triglycerides, FFAs = free fatty acids.

The manifestation of metabolic diseases is linked to predisposition to various risk factors, including aging, genetic background, and chemical exposure [1]. However, the prevailing hypothesis links overnutrition in combination with lifestyle adjustments like physical inactivity and the consumption of a high-fat diet (HFD) with the development of the metabolic syndrome [2]. In this state, the excessive availability of nutrients can have a profound effect in driving metabolic dysregulation in essential body parts, including the skeletal muscle, the adipose tissue, and liver [3]. Excessive fat accumulation in the liver initiates the pathogenesis of non-alcoholic fatty liver disease (NAFLD), along with oxidative stress and inflammation (Figure 1). As such, accumulative research has focused on understanding the pathophysiological mechanisms of liver injury in conditions of metabolic syndrome, especially those mediated by oxidative stress and an abnormal inflammatory response.

It is now well established that an imbalance between the production and detoxification of free radical species, in favour of oxidative stress, is a prominent feature of metabolic syndrome [4]. The latter describes a combination of metabolic dysregulations such as obesity and dyslipidaemia that collectively increases the risk of type 2 diabetes, cardiovascular disease, and NAFLD [2,5,6]. The excessive hepatic fat accumulation in the absence of any clear reason like alcohol consumption is unique to NAFLD, making this condition one of the leading causes of death worldwide [7,8]. Many studies have linked enhanced markers of oxidative stress with the pathogenesis of NAFLD [4,9,10,11]. However, beyond oxidative stress, inflammation has been another crucial component that influences liver function in NAFLD.

Although inflammation is required for an effective immune response, its dysregulation plays a fundamental role in the propagation of metabolic dysregulations [12]. In NAFLD, an impaired inflammatory response is usually characterized by aberrant levels of cytokines and chemokines that are persistent with enhanced hepatic damage [13]. In such conditions, tenacious activation of innate immune responses, mostly driven by excess hepatic fat storage, can promote the classic features of pathological liver inflammation [14]. For example, the sustained elevation of pro-inflammatory mediators such as interleukin (IL)-6, tumour necrosis factor alpha (TNF-α) and nuclear factor kappa-light-chain-enhancer of activated B cells (NF-κB), and transforming growth factor beta (TGFβ)-1 are likely to accelerate liver fibrosis and cause non-alcoholic steatohepatitis (NASH), a severe condition of NAFLD [14,15,16,17]. Lifestyle interventions such as weight loss and regular exercise have been useful in managing or reversing the early stages of NAFLD [18]. However, there is currently an unmet need of effective regimens to treat NASH. Interestingly, therapeutic interventions against both oxidative stress and inflammation are increasingly recognized as attractive to ameliorate vast metabolic abnormalities, including NASH.

Many researchers, including our group, have focused on screening various pharmacological compounds for their ameliorative properties against the metabolic syndrome [19,20,21,22]. Over the years, it has become apparent that pharmacological interventions with strong antioxidant properties like N-acetyl cysteine (NAC) are essential in attenuating oxidative stress and inflammation in metabolic dysfunction. NAC was developed as a remedy for paracetamol overdose in the early 1970s [23], and its enhanced capacity to replenish hepatocellular glutathione (GSH) levels as well as reduce pro-inflammatory cytokines is evident in experimental models of NAFLD. In a comprehensive analysis of literature published in 2015 [24], it was proposed that NAC can improve liver function by attenuating mediators of oxidative stress and inflammation. Since then, many studies have been published assessing the impact of NAC in ameliorating complications related to the metabolic syndrome. Even though such evidence is accumulating, it has not been critically reviewed to inform the therapeutic effects of this pharmacological compound in protecting against NAFLD or the complications implicated in its aggravation such as oxidative stress and inflammation.

Here, we took a systematic approach to retrieve and critically assess the relevant studies reporting on how NAC impacts the liver function in rodent models of NAFLD or patients with this condition. In addition, we provide a brief background on the prominent pathophysiological mechanisms involved in the development and progression of NAFLD to highlight the therapeutic potential of NAC.

## 2. Methods for Study Inclusion

In this review, we modified the previously published protocol to design a relevant search strategy and study inclusion criteria [25]. In brief, three investigators (P.V.D., B.B.N. and T.M.N.) searched and identified relevant articles through a systematic search of major electronic databases such as MEDLINE (PubMed), Cochrane library, Google scholar and EMBASE. This was done from inception until the end of June 2020. The search strategy was adapted to each database using key words and Medical Subject Headings (MeSH) terms ‘N-acetyl cysteine’, ‘fatty liver disease’, ‘oxidative stress’ and ‘inflammation’. This was done to encompass consistent synonyms and linked terms for each item. The current systematic search was done without any language limitations. The reference list, including the removal of duplicates was done by using EndNote version 10 (Clarivate Analytics, Philadelphia, PA, USA).

The current review included in vivo studies from rodent models of NAFLD reporting on the therapeutic effects of NAC. In addition, we considered ex vivo studies focusing on the primary isolated hepatocytes from animals treated with NAC. We also included clinical studies assessing the therapeutic effects of NAC against NAFLD, with the involvement of patients. To limit the narrative of the review, and to enhance its translational potential, we chose to exclude in vitro studies using immortalized cells or primary hepatocytes treated with NAC. The main outcome of interest was the protective effect of NAC against NAFLD or its linked complications such as oxidative stress and inflammation. Unpublished or ongoing studies including review articles were only checked for primary findings, while editorials, and letters were excluded.

## 3. A Brief Overview on NAFLD and Implicated Pathophysiological Mechanisms

NAFLD is the foremost cause of chronic liver disease that affects almost 25% of the world’s westernized population [8]. In spite of its high prevalence, very few patients are known to develop characteristic features of chronic liver injury such as inflammation and fibrosis, while most of them display simple steatosis [26,27]. Thus, much effort has been made to improve the understanding of the pathophysiological mechanisms implicated in the development of NAFLD and NASH. Emerging mechanisms such as the involvement of the gut–liver axis–link have been discussed [28,29]. However, enhanced hepatic lipid accumulation, oxidative stress and inflammation remain the foremost pillars in the pathogenesis of NAFLD [29].

Enhanced hepatic lipid accumulation due to increased adipose tissue-linked lipolysis, especially in conditions of obesity, remains the hallmark of NAFLD. In such conditions, adipose tissue promotes excess glycerol release and free fatty acid (FFA) flux that saturates the liver at rates that exceed the capacity of mitochondrial β-oxidation [26,27]. Accordingly, the activation of 5′ AMP-activated protein kinase (AMPK) to control mitochondrial β-oxidation is progressively recognized as one of the major systems to regulate cellular energy metabolism in different tissues including the liver [30]. The regulation of β-oxidation by AMPK has been credited to its direct phosphorylation of acetyl-CoA carboxylase or interaction with carnitine palmitoyltransferase 1, thus moderating the supply of long chain free fatty acids into the mitochondria for breakdown in diverse tissues [30]. In the liver, AMPK activation remains instrumental for regulating glucose uptake while blocking cholesterol and triglyceride synthesis [31]. Notably, impaired glucose metabolism in the liver can promote the synthesis of lipid storage from acetyl-CoA through de novo lipogenesis (DNL), as previously discussed [32]. Thus, if not secreted into the blood stream as very-low-density lipoprotein (VLDL), enhanced lipogenesis leads to an alteration in hepatic metabolic pathways and the subsequent development of NAFLD. Importantly, patients with NAFLD can display as much as 26% of lipids derived from hepatic DNL [33]. Experimental models of metabolic syndrome also show that, besides the regulation of MAPK, other transcriptional factors such as peroxisome proliferator-activated receptor gamma (PPARγ), CAAT/enhancer binding protein-α (C/EBPα) and sterol regulatory element binding protein (SREBP)-1c are increasingly studied for their role in adipogenesis and lipid-induced hepatic toxicity [31,34,35]. Thus, the inhibition of de novo lipid synthesis through the effective control of substrate metabolism appears to be a vital strategy to improve cholesterol homeostasis and reverse liver damage in conditions of NAFLD [36].

Metabolic inflexibility as a result of enhanced hepatic lipid synthesis and storage is acknowledged to be one of the major mechanisms responsible for mitochondrial dysfunction and the generation of oxidation stress in conditions of metabolic syndrome [26,27]. One of the prevailing theories suggest that increased hepatic FFA flux can impede the efficiency of the mitochondrial transport chain during energy production, and in the process, cause the excessive production of reactive oxygen species (ROS). Certainly, the overproduction of mitochondrial ROS parallels with the suppression of intracellular antioxidant detoxification responses, including GSH, superoxide dismutase and peroxiredoxins in conditions of NAFLD, as reviewed elsewhere [11,37]. In addition to the chain activation of other detrimental free radical species such as reactive nitrogen species, uncontrolled ROS overproduction can prompt the unfolded protein response in the endoplasmic reticulum, thus accelerating hepatic damage during the progression of NAFLD [11,37]. Even though several studies show an association between the levels of lipid oxidation products and disease progression, experimental evidence suggests that compounds such as reactive aldehydes and cholesterol oxidation products are characteristic features of hepatic oxidative damage [11]. In a multi-hit hypothesis, mediators of both oxidative stress and inflammation are increasingly being targeted to ameliorate complications linked with the development of NASH [36].

**Figure 2:**
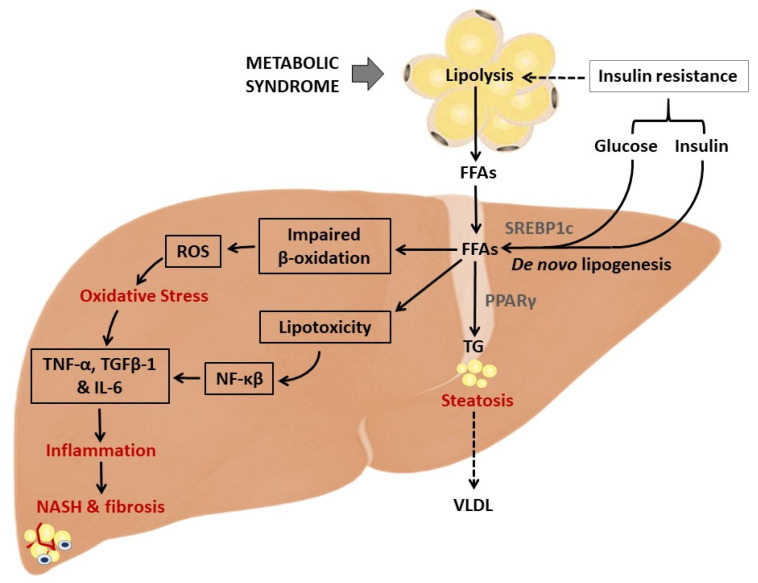
An overview of the pathophysiological mechanisms that implicate oxidative stress and inflammation in the development and worsening of non-alcoholic fatty liver disease (NAFLD). Briefly, in an obese state, adipose tissue can promote excess glycerol release and free fatty acid (FFA) flux that saturates the liver at rates that exceed the capacity of mitochondrial β-oxidation, leading to the activation of transcriptional factors like peroxisome proliferator-activated receptor gamma (PPARγ). In an aggravated state, this process promotes the increased production of reactive oxygen species (ROS) and aberrant levels of pro-inflammation markers such as tumour necrosis factor alpha (TNF-α), the nuclear factor kappa-light-chain-enhancer of activated B cells (NF-κB), and transforming growth factor beta (TGFβ)-1, which are linked with the development of steatosis. ROS = reactive oxygen species; TG = triglycerides; SREBP1c = sterol regulatory element binding protein; NASH = non-alcoholic steatohepatitis; IL-6 = interleukin 6; VLDL = very low density lipoproteins.

In addition to the devastating effects of oxidative stress products, inflammation is one of the prominent causal factors that accelerates liver damage. Briefly, in a state of insulin resistance, excess white adipose tissue can induce lipolysis and the release of adipokines and pro-inflammatory markers such as leptin, TNF-α and IL-6, which contribute to the activation of the inflammasome (Figure 2). The latter describes cytosolic multiprotein oligomers of the innate immune system accountable for the initiation of inflammatory responses [38]. A previous study showed that NLR family pyrin domain containing 3 (NLRP1/3) inflammasome levels are elevated in both parenchymal and nonparenchymal cell types in an HFD-induced mouse model of NASH [39]. Hence, in addition to common pro-inflammatory mediators like IL-6, TNF-α and IL-1β, reducing NLRP3 inflammasome activity can alleviate obesity-associated metabolic abnormalities.

## 4. An Overview of Evidence on the Impact of NAC on NAFLD-Related Complications

A systematic search through major electronic databases identified approximately 43 relevant studies, published between 1997 and 2020, reporting on the impact of NAC in NAFLD-related complications. The subsequent sections describe different effects of NAC in modulating the pathophysiological mechanisms implicated in the development of liver function in preclinical models and human subjects with NAFLD. Particular emphasis is placed on understanding how NAC affects lipid accumulation and the mechanisms involved in oxidative stress and inflammation

### 4.1. NAC Targets Hepatic Lipid Accumulation to Improve Liver Function in Preclinical Models of NAFLD

Table 1 gives an overview of the studies reporting on the impact of NAC on hindering hepatic lipid accumulation to improve liver function in experimental models of NAFLD. Earlier evidence by Lin et al., 2004 [40] demonstrated that NAC at 1 g/L in drinking water for 4 weeks could significantly reduce malic enzyme and fatty acid synthase activities, while lowering triglyceride levels in plasma and liver in HFD-fed mice. In agreement, Samuhasaneeto et al., 2007 [41] showed that NAC supplementation at 230 mg/kg body weight for 8 weeks could reduce serum lipid levels in correlation with reversing the severity of liver histopathological lesions. El-Lakkany et al., 2016 [42] showed that beyond improving serum lipid levels, NAC taken with a diet at 150 mg/kg for 8 weeks could improve the biochemical and histological parameters related to hepatic steatosis such as lobular inflammation and fibrosis, while enhancing liver function enzymes such as serum alanine amino transferase (ALT), aspartate aminotransferase (AST), alkaline phosphatase (ALP), and gamma glutamyl transferase in HFD-induced rats.

Only one study showed limited effects of NAC to reverse HFD-induced liver damage in experimental models of NAFLD (Table 1). Here, Machado et al., 2016 [43] demonstrated that NAC administration at 250 mg/kg body three times a week for 8 weeks failed to inhibit caspase-2 activation in a rodent model of NASH. This could have been related to the severe model of NASH that was used to test the bioactive properties of this antioxidant. However, such a hypothesis has to be confirmed in other similar experimental models. Nonetheless, mechanistically, the summarized evidence supports the notion that NAC could block lipid accumulation by the downregulation of SREBP-1c/SREBP-2 [44] or that of cluster of differentiation 36 (CD36) [45] and PPARγ, while enhancing intracellular antioxidant levels [46] in experimental models of NAFLD.

**Table 1:** An overview of studies on N-acetyl cysteine (NAC) and its impact on hepatic lipid accumulation in preclinical models of NAFLD.

Author, Year	Experimental Model, NAC Dosage, and Intervention Period	Main Findings
Lin et al., 2004 [40]	Male Balb/cA mice consuming a high saturated fat diet (with 18% saturated fat) received NAC (1 g/L) in drinking water for 4 weeks.	NAC significantly reduced malic enzyme and fatty acid synthase activities, and significantly lowered TG levels in the plasma and liver. NAC also reduced cholesterol levels in the plasma and liver and improved high saturated fat diet-related hyperglycaemia, hyperuricemia, and oxidation stress.
Samuhasaneeto et al., 2007 [41]	Male Sprague-Dawley rats fed high fat diet (HFD) for 6 weeks then given 500 mg/kg/day of NAC for 4 weeks.	Treatment with diet or diet plus NAC reduced the levels of cholesterol back to normal. Liver sections from NAC treatment showed a decrease in fat.
Lin and Yin, 2008 [44]	NAFLD was induced through HFD in male C57BL/6 mice, whilst NAC (1 g/L) was directly added into the drinking water as a supplement for 4 weeks.	NAC significantly decreased triacylglycerides and total cholesterol levels via lowering the activity and mRNA expression of lipogenic-related enzymes. NAC also suppressed high saturated fat-induced hepatic mRNA expression of sterol regulatory element-binding protein (SREBP)-1c and SREBP-2.
Korou et al., 2010 [47]	Male C57bl/6 mice received the test diet with NAC supplementation (230 mg/kg body weight) and the high cholesterol group was fed the test diet enriched with 10% sesame oil for 8 weeks.	NAC reduced lipid levels. In terms of liver histology, the lesions observed in the NAC-treated animals were less severe than those seen in the other high cholesterol groups.
Lai et al., 2012 [45]	Male Sprague-Dawley rats fed a modified diet supplemented with 1.0% NAC. After one week, rats on each diet were exposed to 0, 1, or 5 μmol/kg body weight PCB 126 (3,3′,4,4′,5-pentachlorobiphenyl)) by i.p. injection and euthanized 2 weeks later.	NAC resulted in a reduction in hepatocellular lipid in both PCB groups. This effect was confirmed by the gravimetric analysis of extracted lipids. The expression of CD36, a scavenger receptor involved in regulating hepatic fatty acid uptake, was reduced with high-dose PCB treatment but unaltered in PCB-treated rats on an NAC-supplemented diet.
El-Lakkany et al., 2016 [42]	NAFLD was induced by HFD for 12 weeks in male Sprague-Dawley rats before treatment with metformin at a dose of 150 mg/kg, NAC at a dose of 500 mg/kg or metformin for 8 weeks.	NAC or metformin individually improved most of these biochemical and histological parameters related to hepatic steatosis such as lobular inflammation, fibrosis accompanied with elevated serum alanine aminotransferase (ALT), aspartate aminotransferase (AST), alkaline phosphatase (ALP), gamma glutamyl transferase, cholesterol, triglycerides, LDL, VLDL, leptin, TNF-α, and TGF-β1. These improvements were more pronounced in the combination treatment.
Ma et al., 2016 [48]	Six-week-old male C57BL/6 mice fed on chow or HFD were treated with NAC (2 g/L) in drinking water for 11 weeks.	NAC blocked fat mass and the development of obesity reducing HFD-induced macrophage infiltration, and enhanced adiponectin gene expression. NAC oral administration suppressed hepatic lipid accumulation, as evidenced by lower levels of triglyceride and cholesterol in the liver. The beneficial effects are associated with a decrease in hepatic peroxisome proliferator-activated receptor (PPAR)γ and its target gene expression.
Machado et al., 2016 [43]	NASH was induced by methionine–choline deficient diet for 8 weeks. NAC was administered in the drinking water, resulting in an estimated consumption of 250 mg/kg body 3 times a week.	NAC failed to inhibit caspase-2 activation, or improve NASH, normalize pantothenate kinase expression, or restore free CoA levels.
Zhou et al., 2017 [49]	Male Sprague-Dawley fed HFD and received NAC (60 mg/kg) or NAC-activated carbon sustained-release microcapsule (ACNAC; 15, 30 and 60 mg/kg) by gastric perfusion daily for 7 weeks.	ACNAC exhibited different degrees of improvement in various indexes such as reducing the activity of ALT, AST and the content of total cholesterol (TC), TG, LDL-C, increased the content of HDL-C and strengthened dipeptidyl peptidase IV protein expression in the liver cell membrane.
Stojanović et al., 2018 [46]	Wistar rats received methionine (0.8 mmol/kg/day) + NAC (50 mg/kg/day i.p) for 21 days.	Methionine reduced AST, ALT, and ALP activity, whilst the NAC application increased activity of antioxidative enzymes and prevented intensive histological changes in the liver.
Shen et al., 2019 [50]	Male Sprague-Dawley rats received NAC (2.4 mmol/kg) for 3 days before D-galactosamine (400 mg/kg).	NAC reduced liver cholesterol, with fish oil showing a greater attenuating effect than NAC.

### 4.2. NAC Targets Oxidative Stress and Inflammation to Improve Liver Function in Preclinical Models of NAFLD

Table 2 summarizes preclinical evidence reporting on the ameliorative effects of NAC against oxidative stress and inflammation in rodent models of NAFLD. Although models of high-fat or cholesterol diet were used to induce liver abnormalities [41,47,51,52], the majority of included studies employed a methionine–choline-deficient (MCD) diet to assess the impact of oxidative stress and inflammation on NAFLD [17,53,54,55,56]. Interestingly, an MCD diet is a classical dietary model of NASH and progressively used to assess therapeutic effects of various pharmacological compounds in protecting against NAFLD [57]. Subjecting animals to these models for different times, ranging from 1 to 12 months, was consistently related to raised markers of hepatic lipid accumulation, inflammation, and oxidative stress. These markers included IL-6, TNF-α and IL-1β, NF-κB, TGFβ-1, and malondialdehyde (MDA) levels (Table 2). Unfortunately, this consequence was closely associated with liver injury, as demonstrated by increased hepatic levels of Bax and caspase-3, as reported in some studies [58].

In terms of the ameliorative effects of NAC against such complications, the initial evidence from Nakano and colleagues [53,54] showed that NAC at 150 mg/kg body weight was able to significantly improve GSH levels to ameliorate steatotic livers before cold storage. This effect was connected in part by the reversal of hypothermic ischemic–reperfusion injury and the amelioration of oxidative stress in Wistar rats fed an MCD diet. Alternatively, de Oliveira et al., 2006 [55], confirmed that S-nitroso-N-acetylcysteine (SNAC) administration at 1.4 mg/kg/day for 4 weeks was positively correlated with a decrease in the concentration of fatty acid hydroperoxides in liver homogenate of Wistar rats fed an MCD diet. In a similar rat model, Mazo et al., 2013 [17] showed that the administration of SNAC at 1.4 mg/kg daily by gavage for 8 weeks could reduce the collagen-occupied area associated with an upregulation of matrix metalloproteinases-13 and -9 and a downregulation of heat-shock protein-60, tissue inhibitors of metalloproteinase-2, TGFβ-1, and collagen-1α. Uzun et al., 2009 [59] reported that NAC at 100 mg/mL twice a day could enhance the regeneration after partial hepatectomy in rats with NAFLD by enhancing the GSH content while reducing MDA levels. Alternatively, Ali et al., 2016 [56] showed that NAC treatment at 20 mg/kg/d for 28 days could significantly improve levels of ALT, TNF-α, glucose, albumin, MDA, GSH, glutathione-S-transferase in Wistar rats subjected to MCD. This included a reduction in total cholesterol, low-density lipoprotein-c, and leptin levels, when compared with the steatosis control.

In fact, the majority of summarized evidence supports the beneficial effects of NAC in improving liver function by ameliorating markers of oxidative stress and inflammation in preclinical models of NAFLD, especially through the enhancement of intracellular glutathione levels (Table 2).

**Table 2:** An overview of studies on the impact of N-acetyl cysteine (NAC) on oxidative stress and inflammation in preclinical models of NAFLD.

Author, Year	Experimental Model, NAC Dosage, and Intervention Period	Main Findings
Nakano et al., 1997; 1998 [53,54]	NAC was administered in a ready-to-use solution, at 150 mg/kg body weight in Wistar rats fed an MCD diet, through the mesenteric vein 15 min before liver harvest.	Addition of NAC to the liver before cold storage significantly improved glutathione (GSH) levels and ameliorated steatotic livers. This effect was associated in part by the reversal of hypothermic ischemic-reperfusion injury and the amelioration of oxidative stress.
Fusai et al., 2005 [60]	New Zealand White rabbits were fed a high-cholesterol (2%) diet. After, an intravenous infusion of NAC (150 mg/kg of body weight) was administered prior to and during the 6 h reperfusion period.	NAC administration significantly improved portal flow, hepatic microcirculation, bile composition and bile flow after 5 h of reperfusion. NAC administration was also associated with less hepatocellular injury, as indicated by ALT serum activity, and decreased the oxidation of dihydrorhodamine to rhodamine.
de Oliveira et al., 2006 [55]	NAFLD was induced in Wistar male rats by a choline-deficient diet for 4 weeks before the oral administration of *S*-nitroso-*N*-acetylcysteine (SNAC; 1.4 mg/kg/day in comparison to those on PBS solution, NAC solution (7 mg/kg/d) for 4 weeks.	The absence of NAFLD in the SNAC-treated group was positively correlated with a decrease in the concentration of forming reactive fatty acid hydroperoxides in the liver homogenate, compared to the control group, while serum levels of aminotransferases were unaltered.
Samuhasaneeto et al., 2007 [41]	Male Sprague-Dawley rats were fed high fat diet (HFD) for 6 weeks then switched to regular dry rat chow + 20 or 500 mg/kg/day of NAC for 4 weeks.	Treatment with a diet or diet plus NAC reduced the levels of GSH, cholesterol, and hepatic MDA back to normal. Liver sections from groups 3–5 showed a decrease in fat deposition and necro-inflammation in hepatocytes.
Thong-Ngam et al., 2007 [61]	Male Sprague-Dawley rats were fed HFD plus 20 mg/kg per day of NAC orally for 6 weeks.	NAC treatment improved the level of GSH but did not affect MDA. NAC also led to a decrease in fat deposition and necro-inflammation.
Baumgardner et al., 2008 [51]	NAFLD induced by overfeeding Sprague-Dawley rats with dietary polyunsaturated fat containing 70% corn oil with or without 2 g/kg NAC (intragastric gavage) for 65 days.	NAC prevented many aspects of NAFLD progression by decreasing the development of oxidative stress and subsequent increases in TNF-α but did not block the development of steatosis.
Uzun et al., 2009 [59]	NAFLD in male Wistar rats was induced with a choline-deficient diet for 4 weeks. NAC (100 mg/mL) was administered intraperitoneally at a dosage of 100 mg/kg per day twice a day, at 08:00 and 20:00 h.	NAC enhanced regeneration after partial hepatectomy in rats with NAFLD by enhancing GSH content while reducing MDA levels.
Korou et al., 2010 [47]	Male C57bl/6 mice received the test diet with NAC supplementation (230 mg/kg body weight) and high cholesterol group fed the test diet enriched with 10% sesame oil for 8 weeks.	NAC reduced lipid levels, concomitant to decreasing serum lipid peroxidation and restored nitric oxide bioavailability. In terms of liver histology, the lesions observed in the NAC-treated animals were less severe than those seen in the other high cholesterol groups.
Mazo et al., 2013 [17]	Non-alcoholic steatohepatitis (NASH)-induced in Sprague-Dawley rats fed with a choline-deficient, high trans-fat diet and exposed to diethylnitrosamine for 8 weeks. Animals received SNAC daily by gavage (8.0 μmol/kg = 1.4 mg/kg) during an 8 week period.	SNAC led to a 34.4% reduction in the collagen-occupied area associated with upregulation of matrix metalloproteinases (MMP)-13 and -9 and the downregulation of heat-shock protein (HSP)-60, tissue inhibitors of metalloproteinase-2, transforming growth factor (TGF)β-1, and collagen-1α.
Ali et al., 2016 [56]	NAFLD was induced by feeding male Wistar rats a MCD diet for four cycles before treatment with NAC (20 mg/kg/d), ursodeoxycholic acid + resveratrol, and ursodeoxycholic acid + NAC orally for 28 days.	Resveratrol and NAC administration significantly improved liver index (resveratrol only), alanine transaminase, TNF-α, glucose, albumin, malondialdehyde (MDA), GSH, glutathione-S-transferase, total cholesterol, low-density lipoprotein-c, and leptin levels compared with steatosis control values.
Ma et al., 2016 [48]	Six-week-old male C57BL/6 mice fed a chow or HFD were treated with NAC (2 g/L) in drinking water for 11 weeks.	NAC blocked the fat mass and HFD-induced macrophage infiltration, and enhanced adiponectin gene expression. NAC suppressed hepatic lipid accumulation, as evidenced by lower levels of triglyceride and cholesterol in the liver. The beneficial effects are associated with a decrease in hepatic peroxisome proliferator-activated receptor (PPAR)γ and its target gene expression.
de Oliveira Rosa et al., 2018 [62]	Streptozotocin-induced diabetic Wistar rats received NAC at 25 mg/kg body weight daily, orally via gavage, for 37 days.	NAC improved hyperglycaemia and hypoinsulinemia, as well as reducing serum ALT and urea, hepatic triglycerides accumulation and oxidative stress biomarkers in the diabetic liver, as well as improving hepatic antioxidant enzymes’ activities, especially restoring GSH content.
Shi et al., 2018 [58]	Three-week old male Sprague Dawley rats were given (intragastrically) high-fat diet (HFD) with/without activated carbon-NAC (ACNAC) treatment (20, 40 and 80 mg/kg) for 7 consecutive weeks.	ACNAC supplementation improved liver pathologies and prevented HFD-induced telomere shortening and improved telomerase activity. ACNAC supplementation also increased the expression of B-cell lymphoma 2 (Bcl-2), but reduced that of Bax and caspase-3.
Wang et al., 2018 [16]	Male Sprague-Dawley rats were fed with HFD to produce the NASH model and treated (intraperitoneal injections) with or without *N*,*N*’-diacetylcystine (DiNAC) at 12.5 mg/kg, 25 mg/kg, and 50 mg/kg body for 8 weeks.	DiNAC reduced the levels of ALT and AST. DiNAC alleviated histological injury. Moreover, DiNAC strongly reduced the generation of inflammatory cytokines, such as interleukin-6 (IL-6), TNF-α and interleukin-1β (IL-1β), through nuclear factor kappa B (NF-κB) downregulation.
Tsai et al., 2020 [52]	NAFLD was induced in male C57BL/6 (B6) mice by administering HF diet for 12 months with NAC (10 mM NAC) dissolved in water for 6 or 12 months.	NAC intake for 6 or 12 months decreased liver steatosis. NAC treatment also reduced cellular apoptosis and caspase-3 expression. With regards to endoplasmic reticulum stress, only treatment at 12 months improved/reduced phospho-protein kinase R-like endoplasmic reticulum kinase and activating transcription factor 4 expression.

### 4.3. Impact of NAC on Lipid Accumulation, Oxidative Stress and Inflammation in Knockout Models of NAFLD

Table 3 summarizes evidence on the molecular mechanisms implicated in the ameliorative effects of NAC against lipid accumulation, oxidative stress, and inflammation in knockout models of NAFLD. In the last decade, various genetic and gene-specific animal knockout models have been used to better understand the disease pathophysiological mechanisms of NAFLD (Table 3). Indeed, just like MCD-deficient systems [63], *ob/ob* mice have become increasingly explored to bridge the phenotype gap that connects the preclinical pathophysiology of NAFLD to the human disease [64]. Interestingly, the majority of studies included in Table 3 made use of *ob/ob* mice to interrogate the therapeutic effects of NAC against NAFLD-linked complications. For instance, Laurent et al., 2004 [65] showed that NAC at 150 mg/kg daily for 2 months could reduce lipid oxidation and increase the intracellular and the mitochondrial pool of GSH. Using the same *ob/ob* mouse model of NAFLD, others showed that, NAC at 7.5 mM or SNAC at 1.4 μmol/kg for 4 weeks could reduce liver steatosis. This was shown to occur in part by lowering the markers of inflammation and lipid oxidation, which led to improved mitochondrial function, oxidative phosphorylation and intracellular antioxidant responses [9,66,67,68,69].

Furthermore, using other preclinical knockout models, Kumar et al., 2019 [69] showed that the pre-incubation of primary hepatocytes with 10 mM NAC for 30 min could block the augmenter of liver regeneration (ALR, essential for respiration and vegetative growth-1 (Erv1), growth factor Erv1-like, hepatopoietin) depletion and lipid accumulation in hepatocytes. Chen et al., 2015 [70] demonstrated that NAC at 1 mg/mL for 8 weeks could suppress DNA damage and the metabolic changes in both wild-type and polymerase η defiant mice exposed to an HFD. Lee et al., 2015 [71] showed that NAC at 2 mM for 2 h was effective in suppressing lipogenesis in primary hepatocytes isolated from wild-type but not in the superoxide dismutase 1 knockout cells. Preziosi et al., 2017 [72] observed that NAC intake at 2 g/kg in drinking water for 3 months protected liver-specific β-catenin knockout mice against hepatic steatosis, injury and fibrosis. This consequence was also associated with effective modulation of protein kinase B (AKT), extracellular signal-regulated kinases (ERK), NF-κB and the reappearance of β-catenin. These beneficial effects by NAC administration were consistent with the suppression of fatty liver development, NLRP3 and caspase-1 activity, while improving the hepatic mitochondrial function in MCD/catalase knockout mice [63,73].

### 4.4. Clinical Evidence on the Impact of NAC on NAFLD-Associated Complications

Table 4 summarizes the evidence on the impact of NAC administration on liver function in human subjects with metabolic syndrome. Currently, very few clinical studies have assessed the impact of NAC on liver function in patients with NAFLD. Available evidence shows that NAC administration at 1 g/day for 3 months significantly improved liver function enzymes such as ALT, AST, and gamma-glutamyl transferase following a 4-week treatment period [74]. Furthermore, intravenous NAC administration, starting at a dose of 20 mg/kg/day and increasing to 10 mg/kg/day every 1 to 2 months, could significantly reduce serum ferritin and liver biochemistries. This is in addition to improving red blood cell GSH levels in three children with parenteral nutrition-induced liver disease [75]. Others showed that the NAC intake at 1.2 g for 12 months could reduce the degree of steatosis and NAFLD activity score when combined with metformin [76]. Moreover, NAC at 5 mM significantly improved the viability of human hepatocytes from a steatotic donor [77]. However, NAC could reduce elevated levels of ALT in patients with NASH after 3 months’ intake at 0.6 g per 12 h [78]. In addition, the use of NAC at 1.8 g/day orally for 5–6 weeks could improve glucose tolerance and peripheral insulin sensitivity. However, this antioxidant could not affect hepatic insulin levels in patients with polycystic ovary syndrome (PCOS) [79]. Although the prevalence of NAFLD is recorded in patients with PCOS [80], additional studies are still required to assess how NAC affects liver function in patients with PCOS.

## 5. Conclusions

Undoubtedly, NAFLD has become the most common liver disease influencing the quality of life of those affected [8]. The rapid rise in patterns of lifestyle modifications such as a sedentary lifestyle and excessive consumption of diets rich in saturated fats has been closely associated with the development of metabolic diseases, including NAFLD [5,29]. Overwhelming literature, also briefly summarized in the current study, has extensively reviewed the pathophysiological mechanisms driving the onset of NAFLD [5,29,38,64]. Apparently, increased energy availability in conditions of metabolic syndrome can directly promote hepatic lipid accumulation, leading to the generation of oxidative stress and an undesirable inflammatory response [5,29,38,64]. Of major concern, while the human body is designed to fend off such undesirable metabolic effects, the intracellular response systems such as antioxidants are also suppressed in conditions of NAFLD [81,82]. Importantly, a reduction in one of the major antioxidants within the human body such as GSH has been consistent with the progression from NAFLD to NASH in some patients [83]. Hence, GSH is an increasing target of pharmacological compounds that have the capacity to enhance intracellular antioxidant defence systems [19,20,21,22].

**Figure 3:**
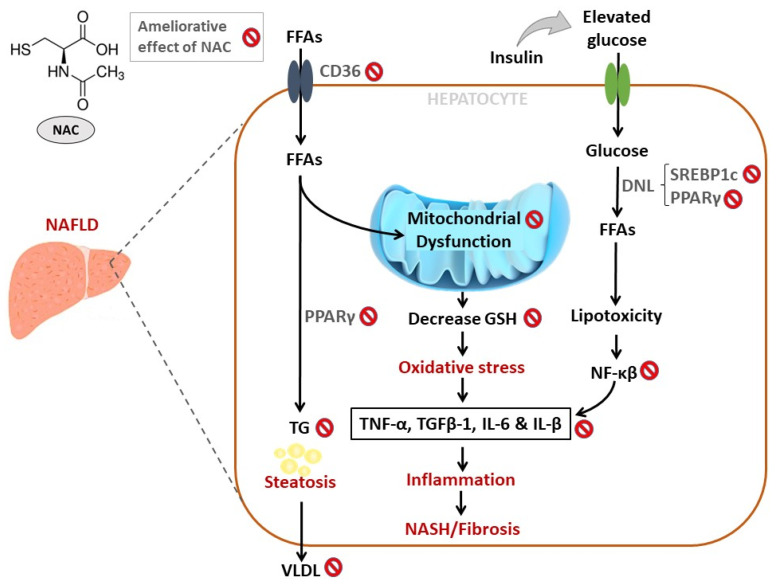
An overview of therapeutic mechanisms linked with the ameliorative effect of n-acetyl cysteine (NAC) against the complications involved in the development of non-alcoholic fatty liver disease (NAFLD). In brief, NAC blocks hepatic lipid accumulation in part through the effective regulation of transcriptional factors such as sterol regulatory element-binding protein (SREBP)-1c/-2 and peroxisome proliferator-activated receptor gamma (PPARγ) in preclinical models of NAFLD. Importantly, NAC appears effective in improving liver function by reducing pro-inflammatory markers such as interleukin (IL)-6, IL-1β, tumour necrosis factor alpha (TNF-α), transforming growth factor beta (TGFβ)-1 and nuclear factor kappa-light-chain-enhancer of activated B cells (NF-κB). This was primarily through the attenuation of lipid peroxidation, improvements in mitochondrial function, and enhancements in intracellular response antioxidants, particularly glutathione (GSH). Abbreviations: TG = triglycerides; DNL = *de novo* lipogenesis.

Overwhelming evidence summarized in the current review supports the beneficial effects of NAC to ameliorate complications linked with the development of NAFLD (Table 1, Table 2, Table 3 and Table 4). In particular, NAC may reverse the severity of liver injury (NASH) in preclinical models of NAFLD by mainly blocking lipid accumulation, through the effective regulation of FFA signaling molecules and transcriptional factors such as SREBP-1c/-2 [44], CD36 [45] and PPARγ [48]. In addition, NAC shows an enhanced effect to improve liver function by reducing the levels of oxidative stress and pro-inflammatory markers such as IL-6, TNF-α and IL-1β, NF-κB, TGFβ-1, and MDA levels (Table 2, Figure 3). This appears to be mainly modulated through the attenuation of lipid peroxidation and enhancements in intracellular response antioxidants, mainly GSH content [41,56,79,80,81,82,83]. Although there are very few clinical studies published on the topic, the evidence summarized in Table 4 suggests that daily intake of NAC at doses between 0.6–1 g is able to improve liver enzymes such as ALT and AST in patients with NAFLD. Thus, well organized randomized clinical trials are still needed to reveal how NAC supplementation affects liver function in individuals with NAFLD. Apparently, due to its safety profile [84], and the rapid growth in published preclinical data informing on its therapeutic mechanisms [85,86,87], there is an increasing interest in developing and validating NAC as remedy for diverse metabolic disturbances. In fact, in addition to clinical trials underway [88], recently reviewed evidence predicts a rapid growth and demand for NAC in global markets, which is an estimated annual growth rate of about 22% over the following years [89].

## Figures and Tables

**Table 3:** An overview of the literature on the impact of NAC on lipid accumulation, oxidative stress and inflammation in knockout models of NAFLD.

Author, Year	Experimental Model, NAC Dosage, and Intervention Period	Main Findings
Laurent et al., 2004 [65]	Male C57BL/6J obese *ob/ob* and lean wild-type mice between 8 and 10 weeks of age fed a standard diet were injected with NAC at 150 mg/kg every 3 days for 2 months.	NAC reduced lipoperoxidation and increased the intracellular and the mitochondrial pool of GSH, whereas the other antioxidant molecules tested were ineffective. However, it failed to ameliorate or lower cytochrome C release and caspase-3 activity.
de Oliveira et al., 2006 [67]	NAFLD was induced in *ob/ob* by MCD diet before oral gavage with SNAC 1.4 μmol/kg) for 4 weeks.	No significant changes in food intake or body weight were observed in comparison to the control group. After SNAC treatment, several genes belonging to oxidative phosphorylation, fatty acid biosynthesis, fatty acid metabolism and glutathione metabolism pathways were downregulated in comparison to the MCD group.
Oliveira et al., 2007 [68]	NAFLD was induced in male *ob/ob* mice using a MCD diet concomitantly with oral SNAC (1.4 mmol/kg) by gavage daily for 4 weeks.	SNAC markedly reduced liver steatosis, as well as parenchymal inflammation and microsomal triglyceride transfer protein.
de Oliveira et al., 2008 [66]	NAFLD was induced in male *ob/ob* mice by MCD diet and high-fat diets before the oral administration of SNAC solution (1.4 mg/kg/day) for 4 weeks.	SNAC inhibited the development of NAFLD, leading to a marked decrease in macro and microvacuolar steatosis and hepatic lipid peroxidation in the MCD group. SNAC treatment reversed the development of NAFLD in animals treated for 60 days with MCD and high-fat diets.
Chen et al., 2015 [70]	Polymerase η defiant (pol η−/−) mice fed HFD received NAC (1 mg/mL; wt/vol) or metformin (1.5 mg/mL; wt/vol) in drinking water, from week 8.	NAC and metformin suppressed DNA damage and the metabolic changes induced by a high-fat diet in both WT and pol η−/− mice, suggesting that metabolic abnormalities may be a general response to elevated DNA damage.
Gentric et al., 2015 [9]	Primary hepatocytes from *ob/ob* mice were treated with 7.5 mM NAC after seeding and during the whole culture time.	NAC treatment impaired the accumulation of ROS as well as that of Gpx3 and Hba1 mRNA, with no impact on cell viability. Moreover, the accumulation of BrdU at 60 h after plating was lower in *ob/ob* hepatocytes treated with NAC than in untreated cells, showing that treated cells progressed normally through the cell cycle.
Lee et al., 2015 [71]	Primary cultured hepatocytes from superoxide dismutase 1 (Sod1)-deficient and wild-type C57BL/6 mice treated with NAC at 2 mM for 2 h.	NAC was found to be effective in suppressing lipogenesis in the WT cells but not in the Sod1-KO cells.
Preziosi et al., 2017 [72]	Iron overload in liver-specific β-catenin knockout mice. NAC (2 g/kg) was added to drinking water for 3 months.	NAC protected KO +Fe from hepatic steatosis, injury and fibrosis, and prevented activation of AKT, ERK, NF-kB and reappearance of β-catenin.
Kumar et al., 2019 [69]	Primary hepatocytes from augmenter of liver regeneration (ALR) (ALR-L-knockout (KO)) mice pre-incubated with 10 mM NAC for 30 min.	NAC and recombinant ALR (rALR) both inhibited ALR depletion-induced miR-540 expression and lipid accumulation in hepatocytes.
Shin et al., 2019 [73]	Male catalase knockout (CKO) mice were fed an HFD for 6 weeks. NAC (60 mg/kg/day) or melatonin (500 μg/kg/day) were dissolved in saline solution and injected intraperitoneally for 6 weeks.	Co-treatment with NAC and melatonin suppressed fatty liver development and improved hepatic mitochondrial function.
Mai et al., 2020 [63]	NAC (10 μM) was applied in combination with MCD/lipopolysaccharide (LPS) or palmitate for 24 h in AML12 (alpha mouse liver 12) cells.	Thioredoxin interacting protein, Nod-like receptor family pyrin domain containing 3 (NLRP3), pro-caspase 1, and caspase-1 activity were suppressed by NAC.

**Table 4:** Clinical studies reporting on the impact of N-acetyl cysteine (NAC) on liver function in conditions of metabolic syndrome and NAFLD.

Author, Year	Experimental Model, NAC Dosage, and Intervention Period	Main Findings
Fulghesu et al., 2002 [79]	Women with polycystic ovary syndrome (PCOS) received NAC at a dose of 1.8 g/day orally for 5–6 weeks.	NAC improved glucose tolerance and peripheral insulin sensitivity, but did not affect hepatic insulin extraction.
Pamuk et al., 2003 [74]	Patients with non-alcoholic steatohepatitis (NASH) received NAC at 1 g/day for 3 months.	NAC significantly improved liver function enzymes such as ALT, AST, and gamma-glutamyl transferase following a 4-week treatment period.
Mager et al., 2008 [75]	Two infants and 1 child with parenteral nutrition-induced liver disease received NAC intravenously, starting at a dose of 20 mg/kg/day and increasing to 10 mg/kg/day every 1 to 2 months. Maximum dosages of NAC of 70 mg/kg/day (patient 3) and 120 mg/kg/day (patient 2) were administered.	All of the patients studied demonstrated significant reductions in serum ferritin and in liver biochemistries when given NAC supplementation IV. In addition, red blood cell GSH levels returned to normal with NAC supplementation in 1 patient.
Khoshbaten et al., 2010 [78]	Patients with non-alcoholic fatty liver steatosis received NAC at 600 mg per 12 h. Patients were followed up using the same method of evaluation repeated in the first, second and third months.	NAC resulted in a significant decrease in serum ALT after three months. This effect was independent of the grade of steatosis in the initial diagnosis.
Sagias et al., 2010 [77]	Hepatocytes from steatotic donor livers were treated with NAC at 5 mM.	Addition of NAC during isolation of human hepatocytes from steatotic donor liver tissue significantly improved the outcome of cell isolation.
Oliveira et al., 2019 [76]	Patients with biopsy-proven NASH, treated with NAC (1.2 g), metformin (850–1500 mg/day) or NAC (1.2g) + metformin (850–1500 mg/day) for 48 weeks.	The combination of NAC and metformin performed better at improving the degree of steatosis, NAFLD activity score, and ALT levels at the end of the treatment.

## References

[1] Heindel J.J., Blumberg B., Cave M., Machtinger R., Mantovani A., Mendez M.A., Nadal A., Palanza P., Panzica G., Sargis R. (2017). Metabolism disrupting chemicals and metabolic disorders. Reprod. Toxicol..

[2] Grundy S.M. (2016). Overnutrition, ectopic lipid and the metabolic syndrome. J. Investig. Med..

[3] McMorrow A.M., Connaughton R.M., Lithander F.E., Roche H.M. (2015). Adipose tissue dysregulation and metabolic consequences in childhood and adolescent obesity: Potential impact of dietary fat quality. Proc. Nutr. Soc..

[4] Hutcheson R., Rocic P. (2012). The metabolic syndrome, oxidative stress, environment, and cardiovascular disease: The great exploration. Exp. Diabetes Res..

[5] Paschos P., Paletas K. (2009). Non alcoholic fatty liver disease and metabolic syndrome. Hippokratia.

[6] Tune J.D., Goodwill A.G., Sassoon D.J., Mather K.J. (2017). Cardiovascular consequences of metabolic syndrome. Transl. Res..

[7] Saklayen M.G. (2018). The global epidemic of the metabolic syndrome. Curr. Hypertens. Rep..

[8] Sherif Z.A., Saeed A., Ghavimi S., Nouraie S.M., Laiyemo A.O., Brim H., Ashktorab H. (2016). Global epidemiology of nonalcoholic fatty liver disease and perspectives on US minority populations. Dig. Dis. Sci..

[9] Gentric G., Maillet V., Paradis V., Couton D., L’Hermitte A., Panasyuk G., Fromenty B., Celton-Morizur S., Desdouets C. (2015). Oxidative stress promotes pathologic polyploidization in nonalcoholic fatty liver disease. J. Clin. Investig..

[10] Hwang I., Uddin M.J., Pak E.S., Kang H., Jin E.J., Jo S., Kang D., Lee H., Ha H. (2020). The impaired redox balance in peroxisomes of catalase knockout mice accelerates nonalcoholic fatty liver disease through endoplasmic reticulum stress. Free Radic. Biol. Med..

[11] Ore A., Akinloye O.A. (2019). Oxidative stress and antioxidant biomarkers in clinical and experimental models of non-alcoholic fatty liver disease. Medicina.

[12] Tilg H., Moschen A.R. (2010). Evolution of inflammation in nonalcoholic fatty liver disease: The multiple parallel hits hypothesis. Hepatology.

[13] Hammerich L., Tacke F. (2014). Interleukins in chronic liver disease: Lessons learned from experimental mouse models. Clin. Exp. Gastroenterol..

[14] Van Herck M.A., Weyler J., Kwanten W.J., Dirinck E.L., De Winter B.Y., Francque S.M., Vonghia L. (2019). The differential roles of T cells in non-alcoholic fatty liver disease and obesity. Front. Immunol..

[15] Su S.B., Chen W., Huang F.F., Zhang J.F. (2018). Elevated Th22 cells correlated with Th17 cells in patients with high liver stiffness in nonalcoholic fatty liver disease. Eur. J. Inflamm..

[16] Wang F., Liu S., Zhuang R., Bao J., Shen Y., Xi J., Sun J., Fang H. (2018). *N*,*N*′-diacetylcystine ameliorates inflammation in experimental non-alcoholic steatohepatitis by regulating nuclear transcription factor kappa B activation. Int. J. Clin. Exp. Pathol..

[17] Mazo D.F., de Oliveira M.G., Pereira I.V., Cogliati B., Stefano J.T., de Souza G.F., Rabelo F., Lima F.R., Ferreira Alves V.A., Carrilho F.J. (2013). S-nitroso-N-acetylcysteine attenuates liver fibrosis in experimental nonalcoholic steatohepatitis. Drug Des. Devel. Ther..

[18] Adams L.A., Anstee Q.M., Tilg H., Targher G. (2017). Non-alcoholic fatty liver disease and its relationship with cardiovascular disease and other extrahepatic diseases. Gut.

[19] Bastin A.J., Davies N., Lim E., Quinlan G.J., Griffiths M.J. (2016). Systemic inflammation and oxidative stress post-lung resection: Effect of pretreatment with N-acetylcysteine. Respirology.

[20] De Rosa S.C., Zaretsky M.D., Dubs J.G., Roederer M., Anderson M., Green A., Mitra D., Watanabe N., Nakamura H., Tjioe I. (2000). N-acetylcysteine replenishes glutathione in HIV infection. Eur. J. Clin. Investig..

[21] Dludla P.V., Dias S.C., Obonye N., Johnson R., Louw J., Nkambule B.B. (2018). A Systematic review on the protective effect of n-acetyl cysteine against diabetes-associated cardiovascular complications. Am. J. Cardiovasc. Drugs.

[22] Dludla P.V., Orlando P., Silvestri S., Mazibuko-Mbeje S.E., Johnson R., Marcheggiani F., Cirilli I., Muller C.J.F., Louw J., Obonye N. (2019). N-Acetyl cysteine ameliorates hyperglycemia-induced cardiomyocyte toxicity by improving mitochondrial energetics and enhancing endogenous Coenzyme Q9/10 levels. Toxicol. Rep..

[23] Bateman D.N., Dear J.W. (2019). Acetylcysteine in paracetamol poisoning: A perspective of 45 years of use. Toxicol. Res..

[24] de Andrade K.Q., Moura F.A., dos Santos J.M., de Araújo O.R., de Farias Santos J.C., Goulart M.O. (2015). Oxidative stress and inflammation in hepatic diseases: Therapeutic possibilities of n-acetylcysteine. Int. J. Mol. Sci..

[25] Dludla P.V., Nkambule B.B., Dias S.C., Johnson R. (2017). Cardioprotective potential of N-acetyl cysteine against hyperglycaemia-induced oxidative damage: A protocol for a systematic review. Syst. Rev..

[26] Nassir F., Rector R.S., Hammoud G.M., Ibdah J.A. (2015). Pathogenesis and prevention of hepatic steatosis. Gastroenterol. Hepatol..

[27] Vuppalanchi R., Chalasani N. (2009). Nonalcoholic fatty liver disease and nonalcoholic steatohepatitis: Selected practical issues in their evaluation and management. Hepatology.

[28] Arab J.P., Arrese M., Shah V.H. (2020). Gut microbiota in non-alcoholic fatty liver disease and alcohol-related liver disease: Current concepts and perspectives. Hepatol. Res..

[29] Buzzetti E., Pinzani M., Tsochatzis E.A. (2016). The multiple-hit pathogenesis of non-alcoholic fatty liver disease (NAFLD). Metabolism.

[30] Garcia D., Shaw R.J. (2017). AMPK: Mechanisms of cellular energy sensing and restoration of metabolic balance. Mol. Cell.

[31] Farmer S.R. (2006). Transcriptional control of adipocyte formation. Cell Metab..

[32] Nagle C.A., Klett E.L., Coleman R.A. (2009). Hepatic triacylglycerol accumulation and insulin resistance. J. Lipid Res..

[33] Donnelly K.L., Smith C.I., Schwarzenberg S.J., Jessurun J., Boldt M.D., Parks E.J. (2005). Sources of fatty acids stored in liver and secreted via lipoproteins in patients with nonalcoholic fatty liver disease. J. Clin. Investig..

[34] Wu S., Lu Q., Ding Y., Wu Y., Qiu Y., Wang P., Mao X., Huang K., Xie Z., Zou M.H. (2019). Hyperglycemia-driven inhibition of AMP-activated protein kinase alpha2 induces diabetic cardiomyopathy by promoting mitochondria-associated endoplasmic reticulum membranes in vivo. Circulation.

[35] Mazibuko-Mbeje S.E., Dludla P.V., Roux C., Johnson R., Ghoor S., Joubert E., Louw J., Opoku A.R., Muller C.J.F. (2019). Aspalathin-enriched green rooibos extract reduces hepatic insulin resistance by modulating PI3K/AKT and AMPK pathways. Int. J. Mol. Sci..

[36] Arguello G., Balboa E., Arrese M., Zanlungo S. (2015). Recent insights on the role of cholesterol in non-alcoholic fatty liver disease. Biochim. Biophys. Acta.

[37] García-Ruiz C., Fernández-Checa J.C. (2018). Mitochondrial Oxidative Stress and Antioxidants Balance in Fatty Liver Disease. Hepatol. Commun..

[38] Wan X., Xu C., Yu C., Li Y. (2016). Role of NLRP3 Inflammasome in the Progression of NAFLD to NASH. Gastroenterol. Hepatol..

[39] Zhang N.P., Liu X.J., Xie L., Shen X.Z., Wu J. (2019). Impaired mitophagy triggers NLRP3 inflammasome activation during the progression from nonalcoholic fatty liver to nonalcoholic steatohepatitis. Lab. Investig..

[40] Lin C.C., Yin M.C., Hsu C.C., Lin M.P. (2004). Effect of five cysteine-containing compounds on three lipogenic enzymes in Balb/cA mice consuming a high saturated fat diet. Lipids.

[41] Samuhasaneeto S., Thong-Ngam D., Kulaputana O., Patumraj S., Klaikeaw N. (2007). Effects of N-acetylcysteine on oxidative stress in rats with non-alcoholic steatohepatitis. J. Med. Assoc. Thai..

[42] El-Lakkany N.M., Seif El-Din S.H., Sabra A.A., Hammam O.A., Ebeid F.A. (2016). Co-administration of metformin and N-acetylcysteine with dietary control improves the biochemical and histological manifestations in rats with non-alcoholic fatty liver. Res. Pharm. Sci..

[43] Machado M.V., Kruger L., Jewell M.L., Michelotti G.A., Pereira Tde A., Xie G., Moylan C.A., Diehl A.M. (2016). Vitamin B5 and n-acetylcysteine in nonalcoholic steatohepatitis: A preclinical study in a dietary mouse model. Dig. Dis. Sci..

[44] Lin C.C., Yin M.C. (2008). Effects of cysteine-containing compounds on biosynthesis of triacylglycerol and cholesterol and anti-oxidative protection in liver from mice consuming a high-fat diet. Br. J. Nutr..

[45] Lai I.K., Dhakal K., Gadupudi G.S., Li M., Ludewig G., Robertson L.W., Olivier A.K. (2012). N-acetylcysteine (NAC) diminishes the severity of PCB 126-induced fatty liver in male rodents. Toxicology.

[46] Stojanović M., Todorović D., Šćepanović L., Mitrović D., Borozan S., Dragutinović V., Labudović-Borović M., Krstić D., Čolović M., Djuric D. (2018). Subchronic methionine load induces oxidative stress and provokes biochemical and histological changes in the rat liver tissue. Mol. Cell. Biochem..

[47] Korou L.M., Agrogiannis G., Pantopoulou A., Vlachos I.S., Iliopoulos D., Karatzas T., Perrea D.N. (2010). Comparative antilipidemic effect of N-acetylcysteine and sesame oil administration in diet-induced hypercholesterolemic mice. Lipids Health Dis..

[48] Ma Y., Gao M., Liu D. (2016). N-acetylcysteine protects mice from high fat diet-induced metabolic disorders. Pharm. Res..

[49] Zhou H., Shi T., Yan J., Chen X., Liao L., Zhao S., Fang H., Zhuang R. (2017). Effects of activated carbon N-acetylcysteine sustained-release microcapsule on dipeptidyl peptidase IV expression in young rats with non-alcoholic fatty liver disease. Exp. Ther. Med..

[50] Shen Y., Lau-Cam C.A. (2019). Taurine enhances the protective actions of fish oil against D-galactosamine-induced metabolic changes and hepatic lipid accumulation and injury in the rat. Taurine 11, Advances in Experimental Medicine and Biology.

[51] Baumgardner J.N., Shankar K., Hennings L., Albano E., Badger T.M., Ronis M.J. (2008). N-acetylcysteine attenuates progression of liver pathology in a rat model of nonalcoholic steatohepatitis. J. Nutr..

[52] Tsai C.C., Chen Y.J., Yu H.R., Huang L.T., Tain Y.L., Lin I.C., Sheen J.M., Wang P.W., Tiao M.M. (2020). Long term N-acetylcysteine administration rescues liver steatosis via endoplasmic reticulum stress with unfolded protein response in mice. Lipids Health Dis..

[53] Nakano H., Nagasaki H., Barama A., Boudjema K., Jaeck D., Kumada K., Tatsuno M., Baek Y., Kitamura N., Suzuki T. (1997). The effects of N-acetylcysteine and anti-intercellular adhesion molecule-1 monoclonal antibody against ischemia-reperfusion injury of the rat steatotic liver produced by a choline-methionine-deficient diet. Hepatology.

[54] Nakano H., Nagasaki H., Yoshida K., Kigawa G., Fujiwara Y., Kitamura N., Kuzume M., Takeuchi S., Sasaki J., Shimura H. (1998). N-acetylcysteine and anti-ICAM-1 monoclonal antibody reduce ischemia-reperfusion injury of the steatotic rat liver. Transplant. Proc..

[55] de Oliveira C.P., Simplicio F.I., de Lima V.M., Yuahasi K., Lopasso F.P., Alves V.A., Abdalla D.S., Carrilho F.J., Laurindo F.R., de Oliveira M.G. (2006). Oral administration of S-nitroso-N-acetylcysteine prevents the onset of non alcoholic fatty liver disease in rats. World J. Gastroenterol..

[56] Ali M.H., Messiha B.A., Abdel-Latif H.A. (2016). Protective effect of ursodeoxycholic acid, resveratrol, and N-acetylcysteine on nonalcoholic fatty liver disease in rats. Pharm. Biol..

[57] Takahashi Y., Soejima Y., Fukusato T. (2012). Animal models of nonalcoholic fatty liver disease/nonalcoholic steatohepatitis. World J. Gastroenterol..

[58] Shi T., Yang X., Zhou H., Xi J., Sun J., Ke Y., Zhang J., Shao Y., Jiang X., Pan X. (2018). Activated carbon N-acetylcysteine microcapsule protects against nonalcoholic fatty liver disease in young rats via activating telomerase and inhibiting apoptosis. PLoS ONE.

[59] Uzun M.A., Koksal N., Kadioglu H., Gunerhan Y., Aktas S., Dursun N., Sehirli A.O. (2009). Effects of N-acetylcysteine on regeneration following partial hepatectomy in rats with nonalcoholic fatty liver disease. Surg. Today.

[60] Fusai G., Glantzounis G.K., Hafez T., Yang W., Quaglia A., Sheth H., Kanoria S., Parkes H., Seifalian A., Davidson B.R. (2005). N-Acetylcysteine ameliorates the late phase of liver ischaemia/reperfusion injury in the rabbit with hepatic steatosis. Clin. Sci..

[61] Thong-Ngam D., Samuhasaneeto S., Kulaputana O., Klaikeaw N. (2007). N-acetylcysteine attenuates oxidative stress and liver pathology in rats with non-alcoholic steatohepatitis. World J. Gastroenterol..

[62] Rosa L.R.O., Kaga A.K., Barbanera P.O., Queiroz P.M., do Carmo N.O.L., Fernandes A.A.H. (2018). Beneficial effects of N-acetylcysteine on hepatic oxidative stress in streptozotocin-induced diabetic rats. Can. J. Physiol. Pharmacol..

[63] Mai W., Xu Y., Xu J., Zhao D., Ye L., Yu G., Wang Z., Lu Q., Lin J., Yang T. (2020). Berberine inhibits Nod-like receptor family pyrin domain containing 3 inflammasome activation and pyroptosis in nonalcoholic steatohepatitis via the ROS/TXNIP Axis. Front. Pharmacol..

[64] Anstee Q.M., Goldin R.D. (2006). Mouse models in non-alcoholic fatty liver disease and steatohepatitis research. Int. J. Exp. Pathol..

[65] Laurent A., Nicco C., Van Nhieu J.T., Borderie D., Chereau C., Conti F., Jaffray P., Soubrane O., Calmus Y., Weill B. (2004). Pivotal role of superoxide anion and beneficial effect of antioxidant molecules in murine steatohepatitis. Hepatology.

[66] De Oliveira C.P., de Lima V.M., Simplicio F.I., Soriano F.G., de Mello E.S., de Souza H.P., Alves V.A., Laurindo F.R., Carrilho F.J., de Oliveira M.G. (2008). Prevention and reversion of nonalcoholic steatohepatitis in OB/OB mice by S-nitroso-N-acetylcysteine treatment. J. Am. Coll. Nutr..

[67] De Oliveira C.P., Stefano J.T., de Lima V.M., de Sa S.V., Simplicio F.I., de Mello E.S., Correa-Giannella M.L., Alves V.A., Laurindo F.R., de Oliveira M.G. (2006). Hepatic gene expression profile associated with non-alcoholic steatohepatitis protection by S-nitroso-N-acetylcysteine in ob/ob mice. J. Hepatol..

[68] Oliveira C.P., Alves V.A., Lima V.M., Stefano J.T., Debbas V., Sa S.V., Wakamatsu A., Correa-Giannella M.L., de Mello E.S., Havaki S. (2007). Modulation of hepatic microsomal triglyceride transfer protein (MTP) induced by S-nitroso-N-acetylcysteine in ob/ob mice. Biochem. Pharmacol..

[69] Kumar S., Rani R., Karns R., Gandhi C.R. (2019). Augmenter of liver regeneration protein deficiency promotes hepatic steatosis by inducing oxidative stress and microRNA-540 expression. FASEB J..

[70] Chen Y.W., Harris R.A., Hatahet Z., Chou K.M. (2015). Ablation of XP-V gene causes adipose tissue senescence and metabolic abnormalities. Proc. Natl. Acad. Sci. USA.

[71] Lee J., Homma T., Kurahashi T., Kang E.S., Fujii J. (2015). Oxidative stress triggers lipid droplet accumulation in primary cultured hepatocytes by activating fatty acid synthesis. Biochem. Biophys. Res. Commun..

[72] Preziosi M.E., Singh S., Valore E.V., Jung G., Popovic B., Poddar M., Nagarajan S., Ganz T., Monga S.P. (2017). Mice lacking liver-specific β-catenin develop steatohepatitis and fibrosis after iron overload. J. Hepatol..

[73] Shin S.K., Cho H.W., Song S.E., Bae J.H., Im S.S., Hwang I., Ha H., Song D.K. (2019). Ablation of catalase promotes non-alcoholic fatty liver via oxidative stress and mitochondrial dysfunction in diet-induced obese mice. Pflug. Arch..

[74] Pamuk G.E., Sonsuz A. (2003). N-acetylcsyteine in the treatment of non-alcoholic steatohepatitis. J. Gastroenterol. Hepatol..

[75] Mager D.R., Marcon M., Wales P., Pencharz P.B. (2008). Use of N-acetyl cysteine for the treatment of parenteral nutrition-induced liver disease in children receiving home parenteral nutrition. J. Pediatr. Gastroenterol. Nutr..

[76] Oliveira C.P., Cotrim H.P., Stefano J.T., Siqueira A.C.G., Salgado A.L.A., Parise E.R. (2019). N-acetyl cysteine and/or usrsodeoxycholic acid associated with metformin in non-alcoholic steaohepatitis: An open label-label multicenter randomized controlled trial. Arq. Gastroenterol..

[77] Sagias F.G., Mitry R.R., Hughes R.D., Lehec S.C., Patel A.G., Rela M., Mieli-Vergani G., Heaton N.D., Dhawan A. (2010). N-acetylcysteine improves the viability of human hepatocytes isolated from severely steatotic donor liver tissue. Cell Transplant..

[78] Khoshbaten M., Aliasgarzadeh A., Masnadi K., Tarzamani M.K., Farhang S., Babaei H., Kiani J., Zaare M., Najafipoor F. (2010). N-acetylcysteine improves liver function in patients with non-alcoholic Fatty liver disease. Hepat. Mon..

[79] Fulghesu A.M., Ciampelli M., Muzj G., Belosi C., Selvaggi L., Ayala G.F., Lanzone A. (2002). N-acetyl-cysteine treatment improves insulin sensitivity in women with polycystic ovary syndrome. Fertil. Steril..

[80] Vassilatou E. (2014). Nonalcoholic fatty liver disease and polycystic ovary syndrome. World J. Gastroenterol..

[81] Uchida D., Takaki A., Adachi T., Okada H. (2018). Beneficial and paradoxical roles of anti-oxidative nutritional support for non-alcoholic fatty liver disease. Nutrients.

[82] Salomone F., Godos J., Zelber-Sagi S. (2016). Natural antioxidants for non-alcoholic fatty liver disease: Molecular targets and clinical perspectives. Liver Int..

[83] Irie M., Sohda T., Anan A., Fukunaga A., Takata K., Tanaka T., Yokoyama K., Morihara D., Takeyama Y., Shakado S. (2016). Reduced glutathione suppresses oxidative stress in nonalcoholic fatty liver disease. Euroasian J. Hepato Gastroenterol..

[84] Safe I.P., Lacerda M.V.G., Printes V.S., Marins A.F.P., Rabelo A.L.R., Costa A.A., Tavares M.A., Jesus J.S., Souza A.B., Beraldi-Magalhães F. (2020). Safety and efficacy of N-acetylcysteine in hospitalized patients with HIV-associated tuberculosis: An open-label, randomized, phase II trial (RIPENACTB Study). PLoS ONE.

[85] Szewczyk-Golec K., Czuczejko J., Tylzanowski P., Lecka J. (2018). Strategies for modulating oxidative stress under diverse physiological and pathological conditions. Oxid. Med. Cell. Longev..

[86] Dludla P.V., Mazibuko-Mbeje S.E., Nyambuya T.M., Mxinwa V., Tiano L., Marcheggiani F., Cirilli I., Louw J., Nkambule B.B. (2019). The beneficial effects of N-acetyl cysteine (NAC) against obesity associated complications: A systematic review of pre-clinical studies. Pharmacol. Res..

[87] Šalamon S., Kramar B., Marolt T.P., Poljšak B., Milisav I. (2019). Medical and dietary sses of n-acetylcysteine. Antioxidants.

[88] U.S. National Library of Medicine ClinicalTrails.gov. https://www.clinicaltrials.gov/ct2/results?cond=&term=n-acetyl+cysteine&cntry=&state=&city=&dist=.

[89] SBWIRE Global Acetylcysteine Market Size will Grow from US$ 490 Million to US$ 1650 Million by 2024. http://www.sbwire.com/press-releases/global-acetylcysteine-market-revenue-will-grow-at-223-cagr-to-2024-with-us-1650-million-market-size-1142001.htm.

